# Editorial: Functional Nanomaterials in Inflammatory Diseases: From Prevention to Diagnosis and Therapy

**DOI:** 10.3389/fphar.2021.802633

**Published:** 2021-11-26

**Authors:** Colin E. Evans, Hua Jin, Jiang Pi

**Affiliations:** ^1^ Program for Lung and Vascular Biology, Stanley Manne Children’s Research Institute, Ann & Robert H. Lurie Children’s Hospital of Chicago, Chicago, IL, United States; ^2^ Department of Pediatrics, Division of Critical Care, Northwestern University Feinberg School of Medicine, Chicago, IL, United States; ^3^ College of Pharmacy, Institute of Clinical Laboratory Medicine, Guangdong Medical University, Dongguan, China; ^4^ Department of Clinical Immunology, Institute of Laboratory Medicine, Guangdong Provincial Key Laboratory of Medical Molecular Diagnostics, School of Medical Technology, Guangdong Medical University, Dongguan, China

**Keywords:** nanomaterials, inflammatory diseases, diagnosis, therapy, anti-inflammation

Inflammation is a complex process involving multiple immune cell types and the inflammatory response is a key characteristic of many human diseases. Inflammatory diseases include acute disorders such as acute lung injury (ALI) and acute respiratory distress syndrome (ARDS), as well as chronic disorders such as cancer and arteriosclerosis. Clinical outcomes for patients with inflammatory disorders may be improved by the development of novel therapies that specifically and rapidly target the diseased tissue. To this end, novel nanomaterials are being designed to target the inflammatory response for the prevention, diagnosis, and treatment of patients with inflammatory diseases.

A major aim of using nanomaterials in the context of inflammatory diseases is to develop novel diagnostic tests and treatment options for patients with inflammatory diseases, which will help combat these disorders and improve clinical outcomes in these patients. *Frontiers in Pharmacology* recently published a series of articles under the Research Topic, “Functional Nanomaterials in Inflammatory Diseases: From Prevention to Diagnosis and Therapy”. This Research Topic contains five Review articles that summarize the use of advanced nanomaterials in distinct types of inflammatory disorders ranging from cancer to prostatitis and rheumatoid arthritis.

Liu et al. describe the challenges associated with treating prostatitis, including inadequate delivery of therapeutic agents to the prostate, which is located deep in the pelvic cavity (Liu et al.). Advantages of nanomaterial-based treatment strategies that are showcased in this review include improvements in controlled drug release by nanoparticles compared with free drug, and the option of loading more than one therapy into nanoparticle-based drug delivery systems (Liu et al.). Another Review summarizes recent literature suggesting that neuro-inflammation could be targeted by nanomedical therapies in diseases such as Alzheimer’s disease, Parkinson’s disease, and amyotrophic lateral sclerosis (Zhu et al.). These authors explain that the blood-brain barrier hinders drug delivery to microglial cells in the central nervous system, and that nanoparticles can act as vehicles for anti-inflammatory drugs to cross the blood-brain barrier, which in turn inhibit over-activation of microglia and excessive neuro-inflammation (Zhu et al.). The Review article by He et al. focuses on the application of gold nanoparticles in cancer immunotherapy (He et al.). Gold nanocarriers can be used for delivery of anti-tumorigenic therapies including drugs and antibodies, for photothermal therapy, or for a combination of both of these treatment strategies (He et al.). In another of the Review articles, authors describe how nanomedicines targeting macrophages show potential in the treatment of rheumatoid arthritis (Li et al.). In their paper, Li et al. present studies suggesting that the crucial effector cell in the progression of rheumatoid arthritis—the macrophage—can be specifically targeted by nanomaterial-based treatment strategies (Li et al.). Finally, Lin et al. review the use of selenium-based nanoparticles in infectious diseases to highlight the important role of selenium in anti-oxidation and cell viability, along with the enhanced stability and drug encapsulation capacity of selenium nanoparticles (Lin et al.).

As convincingly shown by this series of Review articles, nanoparticle-based treatment strategies are highly promising for the treatment of several inflammatory diseases. However, large-scale clinical studies will be required to determine whether the benefit of such treatment strategies shown in pre-clinical settings will translate to clinical benefit in humans.

Three Original Research articles are also included in the Research Topic. Yang et al. describe the preparation and administration of carboxymethyl chitosan hydrogels that are cross-linked with a thioketal agent and loaded with the reactive oxygen species scavenger, curcumin, in the scenario of wound healing following burn injury (Yang et al.). Authors showed that the cross-linked and drug-loaded hydrogels increased migration and viability in fibroblasts, reduced inflammatory cytokine expression in macrophages, and accelerated wound healing, hair regrowth, and revascularization following burn injury in rats (Yang et al.). While this study did not compare the treatment efficacy of the drug-loaded hydrogels with free drug alone or verify findings in a second model of inflammatory injury, future studies could aim to determine whether such treatment strategies are effective in human wound healing and also assess whether this treatment is effective in other scenarios. The second Original Research article describes the preparation and testing of itraconazole-loaded poly lactic-co-glycolic acid (PLGA) nanoparticles in cultured macrophages (Mejía et al., 2021). The authors studied how nanoparticle-to-drug ratio, aqueous phase pH, and type and concentration of surfactant altered the formation, drug-loading capacity, and encapsulation efficiency of the nanoparticles (Mejía et al., 2021). Future studies could test whether these itraconazole-loaded nanoparticles can be used to effectively treat inflammatory injury in multiple animal models of infection. The impact of the drug-loaded nanoparticles could also be tested on different cell types following infection with different fungal or bacterial strains. The third Original Research article presents a platelet membrane coated, PLGA drug delivery system, to administer berberine to the lungs of mice with experimental asthma (Jin et al.). These authors also show that coating of nanoparticles with a platelet membrane increases nanoparticle accumulation in the asthmatic lung compared with free drug or uncoated drug-loaded nanoparticles (Jin et al.). They also show that coating of berberine-loaded nanoparticles with a platelet membrane reduces inflammatory cell numbers and cytokine expression in asthmatic lungs compared with free drug or uncoated drug-loaded nanoparticles (Jin et al.). While this study did not verify the mechanism of uptake or action of the platelet-coated nanoparticles, the authors do provide circumstantial evidence that the nanoparticles modulate the expression of Th1 versus Th2 type cytokines (Jin et al.). Future studies could assess the efficacy of this nanoparticle drug delivery system in other experimental models of inflammatory lung injury, such as endotoxemia or polymicrobial sepsis mouse models.

In conclusion, different nanomaterials can be leveraged to improve diagnostic testing and therapeutic treatments for a variety of inflammatory disorders. In future, nanomedical advances will hopefully lead to the development of novel therapies that improve clinical outcome in patients with inflammatory diseases.

